# Early Diagnosis of *Mycoplasma pneumoniae* in Children: Simultaneous Amplification and Testing (SAT) Is the Key

**DOI:** 10.3389/fped.2019.00441

**Published:** 2019-10-25

**Authors:** Jieqiong Li, Lin Sun, Xirong Wu, Yan Guo, Weiwei Jiao, Jing Xiao, Baoping Xu, Adong Shen

**Affiliations:** ^1^Beijing Key Laboratory of Pediatric Respiratory Infection Diseases, Key Laboratory of Major Diseases in Children, Ministry of Education, National Clinical Research Center for Respiratory Diseases, National Key Discipline of Pediatrics (Capital Medical University), Beijing Pediatric Research Institute, Beijing Children's Hospital, Capital Medical University, National Center for Children's Health, Beijing, China; ^2^Department of Respiratory Diseases, National Center for Children's Health, Beijing Children's Hospital, Capital Medical University, Beijing, China

**Keywords:** *Mycoplasma pneumoniae* pneumonia, children, simultaneous amplification and testing (SAT), Antibody (Ab) testing, diagnosis

## Abstract

**Objective:** The effective diagnosis of *Mycoplasma pneumoniae* (MP) pneumonia (MPP) in children has been hampered by the difficulty of achieving an early diagnosis. The simultaneous amplification and testing (SAT) has the potential for early diagnosis of MP in children.

**Methods:** Of the 1,180 children enrolled in this study, 169 were MPP antibody (Ab) seroconversion positive, 641 showed MPP positivity with a single Ab test, and 370 were MPP negative. Sera and pharyngeal swabs were collected for antibody testing and SAT detection, respectively, on admission. When the samples were Ab negative, the paired -Ab test was requested for MP 7 days later.

**Results:** Using the Ab results as the diagnostic standard, the sensitivity, specificity, positive predictive values (PPV), and negative predictive values (NPV) for SAT were 72.8, 95.1, 97.0, and 61.5%, respectively. SAT had superior diagnostic value in the MPP group who had undergone Ab seroconversion (sensitivity: 82.2%; NPV: 92.1%) and in the short-course group also (sensitivity: 81.0%; NPV: 81.3%). Good agreement was observed between SAT and the paired-Ab results (kappa value = 0.79; *P* < 0.001), but there was a lack of consistency between SAT and the single-Ab test results on admission (kappa value = 0.54, *P* < 0.001).

**Conclusions:** SAT is a rapid, sensitive, and specific method for MP diagnosis in pediatric patients. Our results indicate its value as an effective diagnostic tool for detecting MPP at the initial stage of an infection.

## Introduction

*Mycoplasma pneumoni*ae (MP), the smallest known free-living organism, is a common cause of upper and lower respiratory tract infections (1). MP causes 20 to 40% of the community acquired pneumonia (CAP) in the general population during epidemics, rising to as much as 70% in closed populations (2). Although MP pneumonia (MPP) is often considered a self-limiting disease, some cases become refractory or fulminant and these cases can have serious extrapulmonary complications, even becoming life threatening in children (3). Therefore, the early and rapid detection of MPP is important for timely clinical treatment.

Traditional diagnostic methods for MP infection in clinical practice include culture and serological analyses. Although culture is the gold standard for MPP diagnosis, this approach is time-consuming and requires specialized media and trained laboratory personnel (4). Antibody (Ab) testing is the main diagnostic tool for MP detection in China. However, a single serologic test, regardless of whether the antibody titer in the sample is high, has limited value for early diagnosis of MPP because IgM antibodies are produced in the early stages of MPP, and persist long after the MP infection has cleared. It was recently reported that short-term serial titration of IgM antibodies could obtain a serological confirmation of MPP by seroconversion or by virtue of elevated IgM titers within a relatively short-period after disease onset. However, use of the paired Ab test has limited early diagnostic utility (5, 6). In addition, the antigenic compositions used in various serological-based diagnostic kits differ among manufacturers. Therefore, the Ab positivity rates determined by each method can differ during the acute stage of a MP infection and the 2–3 weeks thereafter (7).

Polymerase chain reaction (PCR) assays are often used for the rapid diagnosis of MPP, replacing the time-consuming and less sensitive culture methods used in clinical practice. Although PCR is the “new gold standard,” it cannot discriminate MP colonization from a true infection. Many studies have been published on the use of PCR to detect MP-DNA or RNA, including conventional, nested, real-time, multiplex, and isothermal amplification methods (8–10). Among these methods, simultaneous amplification and testing (SAT) is a recently developed method based on isothermal amplification of RNA (11, 12). SAT can be completed in 2–3 h, making it highly suitable for rapid detection of MP (13). It is quicker than conventional culturing and serological analysis and, importantly, it has excellent reproducibility. Notably, because increases in bacterial RNA levels can reflect bacterial multiplication; high levels can reflect the living status of bacteria (14). The SAT was previously established to detect *Mycobacterium tuberculosis* and hepatitis C virus (13, 15, 16). Two research groups have also applied the test for early detection of MP infection and reported its good diagnostic accuracy in pediatric patients with CAP (11, 12). However, these studies were mainly focused on the comparison of SAT with PCR using DNA as the template. As mentioned above, in China, Ab is the major diagnostic tool for MP detection, especially the basic-level hospitals. Thus, for China, comparing SAT with Ab test for MP will be meaningful for clinicians based there.

Hence, this study was performed to provide data on MP-related diagnostic methods by specifically answering the following questions: (1) what is the diagnostic efficiency of SAT in children with MPP, and (2) what are the advantages of SAT for MP diagnosis? Our data provide a comprehensive evaluation of SAT, a method with the potential to improve MPP diagnosis in children.

## Materials and Methods

### Patients

This study was conducted at Beijing Children's Hospital between February 2014 and July 2017. All children diagnosed with CAP, as based on CAP management guidelines, were enrolled. CAP was defined as follows. (1) An acute infection of the lung parenchyma and/or interstitial site. (2) Fever, cough, rapid breathing, dyspnea, and dry or wet rales. (3) The disease was acquired outside a hospital or long-term care facility, occurring within 48 h of hospital admittance, or in a patient presenting with pneumonia who lacks the features of healthcare-associated pneumonia. (4) The presence of abnormal changes in chest X-rays (e.g., lung portal lymph node and lung gate shadows, bronchopneumonia, interstitial pneumonia, and large and high-density shadows) (17).

Sera and pharyngeal swab were collected for Ab detection and SAT respectively, on admission. When the samples were negative for specific Abs, a paired Ab test was requested in 7 days later. The exclusion criteria were as follows: the inability to request SAT on admission; the inability to request Ab testing on admission; and the inability to request paired Ab testing on patients with negative single Ab results.

Pediatric MPP was diagnosed according to the guidelines of the Chinese Medical Association as follows: (1) fever, acute respiratory signs (cough, tachypnea, breathing difficulty); (2) shallow breathing and dry or wet rales; (3) chest film with lung portal lymph node and lung gate shadow, bronchopneumonia, interstitial pneumonia, and large and high-density shadows; (4) positive PCR or antibody test (18, 19). Children with MPP were further divided into “the single Ab positive group (MP antibody titer ≥1:160 on admission)” or “the Ab seroconversion group (MP antibody titer seroconversion from negative to positive)”. Children without MPP were diagnosed with viral or bacterial pneumonia and all had paired-negative Ab results (19). Depending on the period from infection onset to hospitalization (days), the patients were classified as the short-course group (≤7 days) or the long-course group (>7 days). The study population's distribution can be seen in Figure S1.

Using the Ab results as the diagnostic standard, the sensitivity, specificity, positive predictive values (PPVs), and negative predictive values (NPVs) for SAT were calculated.

### Ethics

This research was approved by the Ethics Committee of Beijing Children's Hospital. All methods and experimental protocols in this study were conducted in accordance with the approved protocols and Ethics Committee's existing guidelines.

### Serological Testing of Abs

Ab classes, including IgM and IgG, were determined using the gelatin particle agglutination assay (SERODIA-MYCO II, Japan). All tests were conducted in accordance with the manufacturer's instructions (11, 12).

### SAT for MP RNA Detection

RNA extraction from pharyngeal swabs was performed using magnetic beads in accordance the manufacturer's instructions (Rendu Biotech., Shanghai, China). RNA was eluted in 40 μl of detection reagent (dNTPs, NTPs, buffer, 500 nM each of primers, 250 nM of probes, and the internal control). Next, a 30 μl aliquot of each RNA sample and 10 μl of the enzyme reagents (MLV and T7) were mixed in a final volume of 40 μl. SAT was performed on an ABI 7500 Real-Time PCR system (11).

### Statistical Analysis

Statistical analyses were conducted using SPSS software (version 20.0). A kappa test with the continuity correction was performed to analyze the agreement between the Ab and SAT results. A two-tailed *P*-value of <0.05 was considered to be statistically significant, and a kappa value >0.75 was considered to indicate perfect agreement.

## Results

### Study Population Characteristics

**Figure 1:**
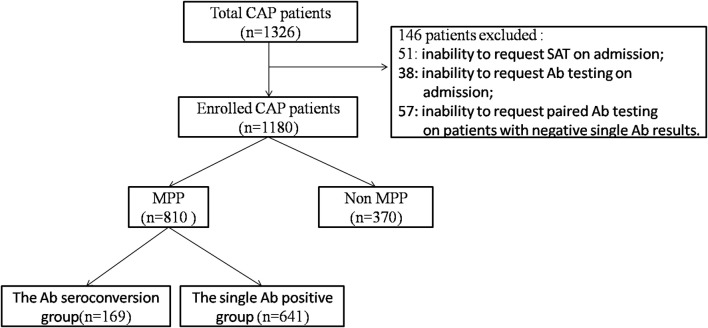
Flow chart of the study design and number of cases analyzed.

A total of 1,326 CAP patients were enrolled in this study. Overall, 146 patients were excluded based on the above-mentioned exclusion criteria (Figure 1). Of the remaining 1,180 patients, 169 (14.3%) were considered to be “Ab seroconversion cases (seroconversion from negative to positive)”; 641 (54.3%) were “single Ab positive cases (MP antibody titer of ≥ 1:160 on admission)”; and 370 (31.4%) were non MPP patients, who were finally diagnosed with bacterial or viral pneumonia. Among all of the patients with MPP, Ab seroconversion in the MPP cases accounted for 20.9% (169/810) and single Ab positive MPP cases accounted for 79.1% (641/810).

The characteristics of participants are shown in Table 1. Patients in the MPP group had a median age of 6.8 years, which was significantly older than that of the patients in the non MPP group (median, 3.8 years; *P* = 0.002). More children in the MPP group had received macrolides before hospital admission compared with the children in the non MPP group (83.3 vs. 54.6%). There was no significant difference in sex between the two groups. Among them, 301 MPP and 220 non MPP patients were in the short-course group (≤7 days), while 509 MPP and 150 non MPP patients were in the long-course group (>7 days).

**Table 1:** Study population characteristics.

**Characteristic**	**MPP (*n* = 810)**	**Non MPP (*n* = 370)**	***P*-value**
Age (y)	6.8 ± 3.1	3.8 ± 3.7	0.002
Male, n (%)	421(51.9)	195(52.7)	0.816
Macrolides therapy, n (%)	675(83.3)	202(54.6)	0.000
Course, n (%)			0.000
≤7 days	301(37.2)	220(59.5)	\
>7 days	509(62.8)	150(40.5)	\

*MPP, Mycoplasma pneumoniae pneumonia*.

### Diagnostic Performance of SAT

Among all of the patients with MPP (single Ab positive cases and Ab seroconversion cases), the sensitivity and specificity of SAT were 72.8% (590/810) and 95.1% (352/370), respectively. The PPV of SAT was 97.0% (590/608) and the NPV was 61.5% (352/872) (Table 2). Taking only the single Ab positive MP cases into account, the sensitivity and NPV of SAT were 70.3% (451/641) and 64.9% (352/542), respectively, whereas in the cases with Ab seroconversion, the sensitivity and NPV of SAT were 82.2% (139/169) and 92.1% (352/382). In contrast, the results showed a clearly higher PPV for SAT in the single Ab positive MPP subgroup than that in the Ab seroconversion MPP subgroup (96.1 vs. 88.5, *P* < 0.001).

**Table 2:** SAT-related sensitivity, specificity, PPV, and NPV.

**Group**	**Sensitivity**	**Specificity**	**PPV**	**NPV**
Total, % (n/N)	72.8 (590/810)	95.1 (352/370)	97.0 (590/608)	61.5 (352/872)
**SUBGROUPS DIVIDED BY AB, % (N/N)**				
Cases with single Ab positive	70.3 (451/641)	95.1 (352/370)	96.1 (451/469)	64.9 (352/542)
Cases with Ab seroconversion	82.2 (139/169)	95.1 (352/370)	88.5 (139/157)	92.1 (352/382)
**SUBGROUPS DIVIDED BY COURSE, % (N/N)**				
Cases with course ≤7 days	81.0 (244/301)	94.5 (208/220)	95.3 (244/256)	81.3 (208/256)
Cases with course >7 days	68.0 (346/509)	96.0 (144/150)	98.3 (346/352)	46.9 (144/307)

*PPV, positive predictive value; NPV, negative predictive value; SAT, simultaneous amplification and testing; MPP, Mycoplasma pneumoniae pneumonia*.

Significantly higher sensitivity and a higher NPV for SAT were also observed in the short-course subgroup unlike the long-course subgroup (sensitivity: 81.0 vs. 68.0%, *P* < 0.001; NPV: 81.3 vs. 46.9%, *P* < 0.001). SAT achieved good specificity and a high PPV in both the short- and long-course subgroups (specificity: 94.5 vs. 96.0%, *P* = 0.523; PPV: 95.3 vs. 98.3%, *P* = 0.057).

### Advantages of SAT for the Diagnosis of MP

#### Better Diagnostic Value in the Short-Course Group and the MP Confirmed Group

To evaluate the diagnostic value of SAT, we compared the positivity rates for SAT and Ab testing for MPP diagnosis on admission (Table 3). In all the patients with MPP (*n* = 810), the Ab positivity rate (79.1%) was higher than that for SAT (72.8%, *P* = 0.003). Among the 169 children with MPP who showed Ab seroconversion, 139 had positive SAT results where; SAT detected an extra 82.2% of the MPP patients with negative Ab results on admission. Interestingly, the SAT positivity rate (81.0%, 244/301) was obviously higher than that of the Ab detection rate (59.8%, 180/301, *P* < 0.001) in the short-course subgroup. Conversely, the Ab test was superior in its diagnostic value for the long-course subgroup than that of SAT (Ab vs. SAT, 90.6 vs. 68%, *P* < 0.001).

**Table 3:** Positivity rates for SAT and Ab testing for MPP diagnosis on admission.

**Group**	**SAT positive rate**	**Ab positive rate**	***P*-value**
Total MPP,% (n/N)	72.8 (590/810)	79.1 (641/810)	0.003
**SUBGROUPS DIVIDED BY AB, % (N/N)**			
Cases with single Ab positive	82.2 (139/169)	0 (0/169)	0.000
Cases with Ab seroconversion	70.3 (451/641)	100 (641/641)	0.000
**SUBGROUPS DIVIDED BY COURSE, % (N/N)**			
Cases with course ≤7 days	81.0 (244/301)	59.8 (180/301)	0.000
Cases with course >7 days	68.0 (346/509)	90.6 (461/509)	0.000

*SAT, simultaneous amplification and testing; Ab, antibody; MPP, Mycoplasma pneumoniae pneumonia*.

#### Good Consistency Between SAT and the Paire Ab Test

The consistency between the SAT and Ab results, including the single Ab and the paired Ab tests were further analyzed. The degree of agreement between SAT and the single Ab test for all the MPP patients was not very high on admission, and 40.6% (329/810) of the samples showed inconsistent SAT and Ab results (kappa value = 0.54, *P* < 0.001). Specifically, 17.1% (139/810) of the cases were SAT(+)/Ab(−) and 23.5% (190/810) were SAT(−)/Ab(+). Among the SAT(+)/Ab(−) cases, 76.2% (106/139) were admitted for shorter than 1 week and the average course was 6.6 days, which was shorter than that for the SAT(−)/Ab(+) cases (17.1 days). Among the SAT(−)/Ab(+) cases, 81.6% were treated with macrolides and the average treatment time was 11.7 days, which was an obviously longer time than for the SAT(+)/Ab(−) cases (3.6 days).

However, the SAT results showed good agreement with the paired-Ab results (kappa value = 0.79; *P* < 0.001). In the Ab seroconversion MPP group, 17.8% (30/169) of these patients were typed as SAT(−)/paired-Ab(+). The remaining 30 patients, who had SAT(−)/paired-Ab(+) results, had a higher rate of macrolide treatment (93.3%, 28/30). Among the non MPP group, 4.9% (18/370) of the patients had SAT(+)/paired-Ab(−) results (Table 4).

**Table 4:** Factors influencing the inconsistent of SAT and Ab results.

**Characteristic**	**Total MPP**		**Ab seroconversed MPP**	**Non MPP**
	**SAT(+)/Ab(−)**	**SAT(−)/Ab(+)**	**SAT(−)/paired-Ab(+)**	**SAT(+)/paired-Ab(−)**
Percentage, % (n/N)	17.1 (139/810)	23.5 (190/810)	17.8 (30/169)	4.9 (18/370)
**HISTORY OF MACROLIDES THERAPY**				
Macrolides therapy, % (n/N)	74.1 (103/139)	81.6 (155/190)	93.3 (28/30)	38.9 (7/18)
Time (day)	3.6 ± 3.5	11.7 ± 21.2	11.3 ± 7.9	6.9 ± 3.9
**COURSE**				
Course ≤7 days, % (n/N)	76.2 (106/139)	22.1 (42/190)	36.6 (11/30)	66.7 (12/18)
Time (day)	6.6 ± 2.8	17.1 ± 13.9	10.7 ± 8.4	3.7 ± 4.3

*MPP, Mycoplasma pneumoniae pneumonia; SAT, simultaneous amplification and testing; Ab, antibody*.

## Discussion

MP is a major cause of pneumonia in children and effective treatment of it requires rapid and accurate detection in clinical practice. In previous studies, Ab results have been reported to be a good indicator of a MP infection. An MP antibody titer of ≥1:160 or a subsequent 4-fold increase is an important standard used for MPP diagnosis (20, 21); however, as a diagnostic tool, this test has several limitations. Therefore, in the present study we evaluated a new molecular assay, SAT, to assess its potential utility for diagnosing pediatric MP infections. An important feature of our study was that SAT and Ab positivity were both evaluated for their ability to detect MPP at the early stage of the disease.

Our data showed that SAT has good diagnostic accuracy in children with MPP, especially those in the MPP group who had undergone with Ab seroconversion, with a sensitivity of 82.2%. The results indicated that SAT has a compatible positivity rate to the paired Ab test. We also observed significantly higher sensitivity and a higher NPV for SAT in the short-course subgroup. It is generally accepted that the production of anti-MP antibodies by the immune system is slow. Children with MPP are admitted to hospital earlier than adults, and older children have more severe clinical signs and symptoms as well as pulmonary lesions (21, 22). Thus, a higher proportion of pediatric patients with MPP had negative Ab results on admission, indicating that the single Ab test is not suitable for the early diagnosis of pediatric MPP in clinical practice. New rapid diagnostic tests such as SAT have potential for improving early clinical diagnosis. However, besides SAT, short-term repeated Ab tests, obtain paired sera within a relatively short-period after the disease onset, may also be necessary to overcome the limitations in the early diagnosis of MP infection (7).

In the consistency analysis of the two tests, only 17.1% of the cases in the MPP group, with an average course of 6.6 days, showed SAT(+)/Ab(−) results on admission. Most children with MPP and inconsistent SAT(+)/Ab(−) results exhibited the clinical characteristics of MPP and responded well to macrolide treatment. The “false-negative” results obtained with the Ab test may be explained by the late appearance of MP antibodies and a weak or deferred antibody response to MP in the young children (23). Additionally, 23.5% of cases had SAT(−)/Ab(+) results on admission, most of which received long macrolide treatment. Thus, one explanation for the “false-negative” SAT results might be this long-term treatment, which may decrease the MP load. The side effects of tetracyclines and quinolones make them unsuitable for use in children. Although macrolide-resistant MP strains might be prevalent in China, macrolides are still the first choice antibiotics because of their beneficial immunomodulatory effects. Our data showed that 81.6% of the cases received macrolide treatment and the average treatment time was 11.7 days before admission to our hospital. Previous data from our laboratory also showed that the SAT positivity rates were significantly lower in the macrolide-treated MPP cases than in the untreated cases with MPP. The second potential explanation for the “false-negative” SAT result is the pathogenesis profile of MP. In the pathogenesis of MP, the acute lung injury that occurs may not necessarily be associated with pathogen-induced cytopathology. Instead, hyper-immune reactions by the host's immune system in response to the infection, as opposed to MP colonization of the upper respiratory tract *per se*, may be sufficient to trigger MPP. Thus, it would not be surprising that many patients with MPP may be PCR negative, especially patients with long-term morbidity and more severe pneumonia, as was found in the present study. The effect of antibiotics on severe MPP may be limited (24). Another potential explanation for the “false-negative” SAT results might relate to the possible poor quality of the swab samples, which require skill to acquire (25), resulting in some samples being below the assay's detection limit (26). After excluding patients with the clinical characteristics of MPP, we determined that the “false-positive” SAT results may be related to MP throat colonization (27). Therefore, it is possible that no single reliable test currently exists for MP infection diagnosis. A combination of various tests is probably the most reliable way of improving the accuracy of diagnosis.

Because antibody responses are delayed during the acute stage of an MP infection, an observable seroconversion in paired Ab titers is considered to be an important standard for MP diagnosis (28). However, difficulty exists with paired serum collection from children, making this approach of limited utility in pediatrics. In the present study, SAT showed better diagnostic potential in the Ab seroconversion MPP group. In total, 139 of 163 patients showed positive SAT results on admission, while their single Ab results were negative. SAT was also shown to be in good agreement with the paired-Ab results. However, in the Ab seroconversion MPP group, 17.8% of the cases still showed SAT(−)/paired-Ab(+) and 93.3% of these cases had been treated with macrolides, which was probably responsible for the negative SAT results. In contrast, in the non MPP group, 4.9% of patients had SAT(+)/paired-Ab(−) results. After excluding patients with the clinical characteristics of MPP, we consider that the “false-positive” SAT results may be attributable to MP throat colonization.

Previously, Li et al. compared SAT with real-time PCR and a high consistency was observed between these two methods (11). Although SAT might have the potential to replace the paired Ab test, it still has several limitations, such as its difficulty in differentiating active infections from MP carriers, its inability to provide definitive evidence of a systemic immune reaction, or the influence of the sampling sites on its diagnostic ability, for example. Thus, combining the use of several methods will, at least for the present time, most likely improve the efficiency of diagnosis.

In conclusion, SAT is a rapid, sensitive, and specific method for MP identification. It may also be an effective and valuable diagnostic tool for clinicians to use to detect MPP during the initial phase of an infection.

## Data Availability Statement

The raw data supporting the conclusions of this manuscript will be made available by the authors, without undue reservation, to any qualified researcher.

## Ethics Statement

The studies involving human participants were reviewed and approved by the Ethics Committee of Beijing Children's Hospital. Written informed consent was collected from the children or the children's parents/guardians. All methods and experimental protocols in this study were conducted in accordance with the approved protocols and Ethics Committee's existing guidelines. Written informed consent to participate in this study was provided by the participants' legal guardian/next of kin.

## Author Contributions

JL, BX, and AS designed the experiments. JL and LS performed the experiments and analyzed the data. XW and WJ provided technical support for the experiments. AS, JX, and YG provided comments and technical advice. JL, LS, BX, and AS wrote the manuscript. All authors have discussed the results, commented on the manuscript, and agreed to be accountable for all aspects of the work in ensuring that questions related to the accuracy or integrity of any part of the work are appropriately investigated and resolved.

### Conflict of Interest

The authors declare that the research was conducted in the absence of any commercial or financial relationships that could be construed as a potential conflict of interest.

## Supplementary Material

Figure S1 Study population distribution. **(A)** Year distribution of MPP and non MPP children. **(B)** Monthly distribution of MPP and non MPP cases. **(C)** Age distribution of MPP and non MPP cases.
