# Supplementary figures and images for: Early Diagnosis of Mycoplasma pneumoniae in Children: Simultaneous Amplification and Testing (SAT) Is the Key
Source: Front Pediatr. 2019 Oct 25;7:441. doi: 10.3389/fped.2019.00441 (PMC6824142; doi:10.3389/fped.2019.00441)

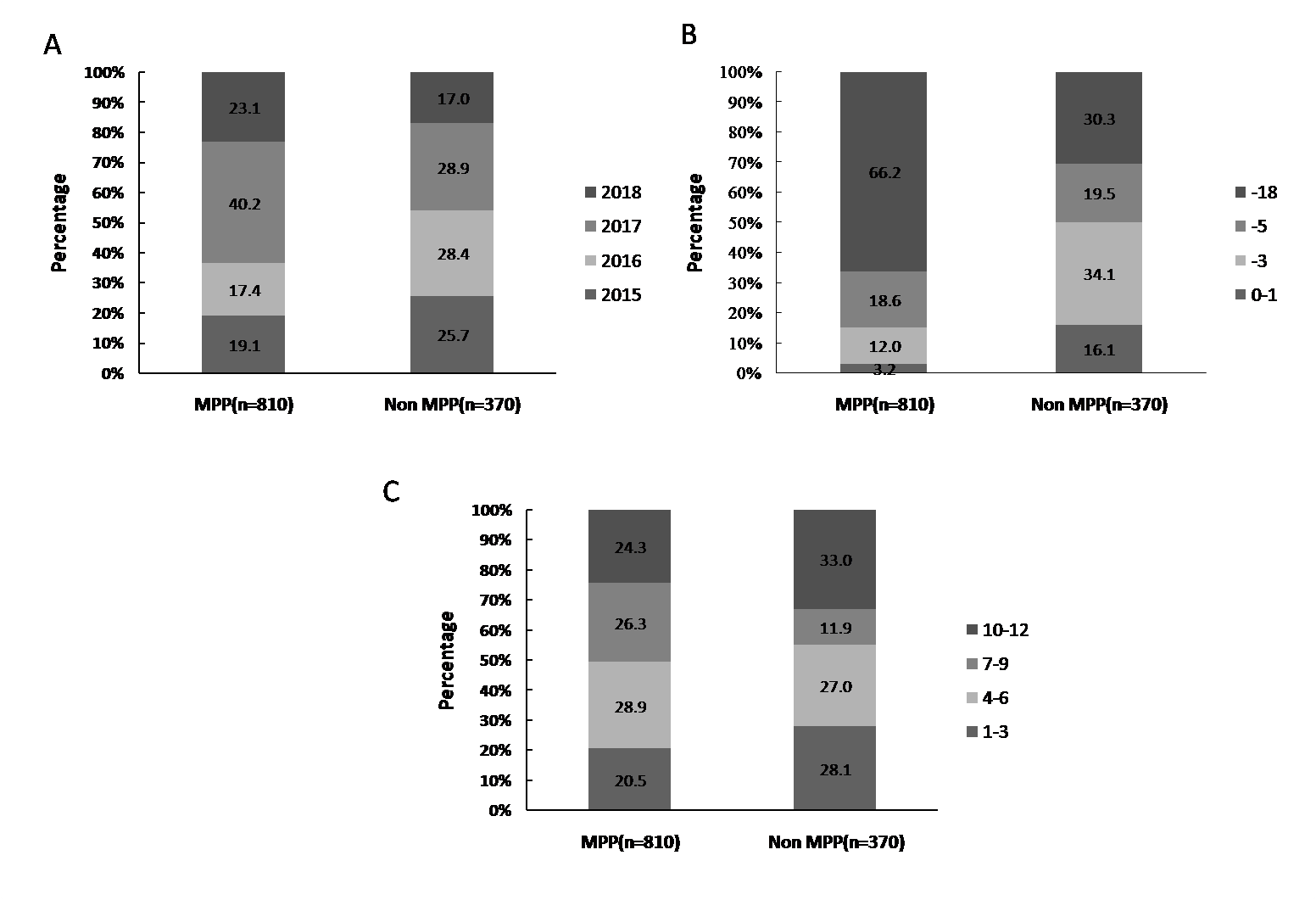

Supplement: Figure S1 — Study population distribution. (A) Year distribution of MPP and non MPP children. (B) Monthly distribution of MPP and non MPP cases. (C) Age distribution of MPP and non MPP cases. [file Image_1.TIF]
